# Potentially Inappropriate Medication at Admission and at Discharge: A Geriatric Study in an Internal Medicine Service in Portugal

**DOI:** 10.3390/ijerph20064955

**Published:** 2023-03-11

**Authors:** Carla Perpétuo, Ana I. Plácido, Jorge Aperta, Adolfo Figueiras, Maria Teresa Herdeiro, Fátima Roque

**Affiliations:** 1 Research Unit for Inland Development, Polytechnic of Guarda (UDI/IPG), 6300-559 Guarda, Portugal; 2 Local Health Unit of Guarda, 6300-035 Guarda, Portugal; 3 Sociedade Portuguesa de Farmacêuticos dos Cuidados de Saúde (SPFCS), Rua D. Manuel I, 74 1° Piso, 3030-320 Coimbra, Portugal; 4 Consortium for Biomedical Research in Epidemiology and Public Health (CIBER en Epidemiología y Salud Pública-CIBERESP), 28029 Madrid, Spain; 5 Health Research Institute of Santiago de Compostela (IDIS), 15786 Santiago de Compostela, Spain; 6 Department of Preventive Medicine and Public Health, University of Santiago de Compostela, 15786 Santiago de Compostela, Spain; 7 Institute of Biomedicine (iBiMED-UA), Department of Medical Sciences, University of Aveiro, 3810-193 Aveiro, Portugal; 8 Health Science Research Center (CICS/UBI), University of Beira Interior, 6201-001 Covilhã, Portugal

**Keywords:** aging, hospital, polypharmacy, potentially inappropriate medicine, Beers criteria, STOPP criteria, EU (7)-PIM list

## Abstract

Aging is associated with an increase in the prevalence of chronic diseases and polypharmacy, and with the prescription of potentially inappropriate medications (PIMs). This study aimed to analyze the variation in PIMs from hospital admission to discharge. A retrospective cohort study was conducted on inpatients of an internal medicine service. According to the Beers criteria, 80.7% of the patients had been prescribed at least one PIM at admission and 87.2% at discharge; metoclopramide was the most-prescribed PIM from admission to discharge, and acetylsalicylic acid was the most-deprescribed one. According to the STOPP criteria, 49.4% of patients had been prescribed at least one PIM at admission and 62.2% at discharge; quetiapine was the most-prescribed PIM from admission to discharge, and captopril was the most-deprescribed one. According to the EU(7)-PIM list, 51.3% of patients had been prescribed at least one PIM at admission and 70.3% at discharge, and bisacodyl was the most-prescribed PIM from admission to discharge and propranolol the most-deprescribed one. It was found that the number of PIMs at discharge was higher than at admission, suggesting the need to develop a guide with adapted criteria to be applied in an internal medicine service.

## 1. Introduction

The world is facing global aging. In 2019, one in eleven people was over 65 years of age, equating to 9% of the total world population [1]. With the increase in the number of older people worldwide, new challenges arise for current societies, namely ensuring that the increase in average life expectancy is accompanied by the maintenance of the quality of life [2]. Physiological alterations associated with age lead to a decrease in the ability to adapt to changes in the external environment, increased susceptibility to disease, and decreased ability to recover, which can cause increased use of healthcare resources, including medicines [3,4,5]. Polypharmacy is quite common in older adults with multiple comorbidities and it is associated with the use of PIMs and the occurrence of potential adverse drug reactions (ADR), but its definition is not consensual; the most common definitions define polypharmacy as the regular use of five or more medications [6].

Renom-Guiteras et al. define PIMs as medicines whose potential risk of occurrence of ADR may outweigh the clinical benefit, especially if there is scientific evidence of safer alternatives [7].

To prevent negative outcomes associated with medication use, several tools have been developed to identify PIMs [8]. The Beers criteria are one of the most known and used tools. They were developed in the United States of America [9], while the Screening Tool of Older Persons’ Prescriptions (STOPP) and the Screening Tool to Alert Doctors to Right Treatment (START) criteria [10] and the EU(7)-PIM list are tools developed in Europe [7].

Although it is known that older inpatients are at particular risk of PIMs, the lack of concordance between the different applied criteria makes the implementation of measures to prevent them difficult [11]. In this context, this study aims to analyze the frequency of PIMs from admission to hospital discharge by applying the Beers 2019 and the STOPP v2 criteria, and the EU (7)-PIM list.

## 2. Materials and Methods

### 2.1. Study Design and Study Population

A retrospective study was conducted in a general internal medicine ward of a first-level hospital located in the center of Portugal. This hospital belongs to NUTS II (nomenclature of territorial units), defined by the center regional administration of health [12]. It covers approximately 51,243 older adults, and the internal medicine ward has a total of 68 beds.

Data were obtained from the hospital’s electronic medical record and included patient age, patient gender (male/female), patient diagnoses (according to the International Statistical Classification of Diseases and Related Health Problems 10th Revision ICD-10), hospitalization days, drugs prescribed, and also medical and laboratory tests, as previously described by Perpetuo et al. [11].

The study population were older patients (aged ≥ 65) hospitalized in the internal medicine ward for at least 4 days during 2019. Older patients hospitalized for less than 4 days were excluded.

The sample size was estimated using the pro package from the statistical software R with an estimated prevalence of 50% and a margin error of 4%. The program reported a sample of 601 patients.

A total of 616 patients were included in the study, for which detailed characterization was previously published [13]. Prescription data were collected on the first day (admission data) and the last day of hospitalization in the internal medicine ward (discharge data).

### 2.2. Data Analysis

All medication prescribed at admission and at discharge, for all the 616 included older inpatients, were analyzed, and the Beers 2019 and the STOPP v2 criteria and the EU(7)-PIM list were applied for the identification of PIMs.

The Beers criteria have been revised several times, with the latest update in 2019. This list includes 30 individual criteria for drugs or drug classes to be avoided in older patients and 16 specific criteria for over 40 drugs or drug classes that should be used with caution or avoided in certain diseases or conditions [14]. In this study, the criteria for screening “Potentially Clinically Important Drug–Drug Interactions That Should Be Avoided in Older Adults” was not applied.

The STOPP/START criteria emerged as a European response to medication-related problems, aiming to identify whether prescribing is appropriate for older patients [10]. These criteria were revised in 2015, and this second version (v2) is organized in terms of physiological systems and presents 114 criteria, of which 80 are STOPP criteria and 34 START criteria [15].

The EU (7)-PIM list was developed through the consensus technique among experts from seven European countries: Estonia, Finland, France, Germany, the Netherlands, Spain, and Sweden. It aims to allow the identification and comparison of prescription profiles of PIMs in older people belonging to the European community, and to develop common indicators allowing the monitoring of medicines. The list comprises 275 active substances and 7 drug classes, belonging to 55 therapeutic classes and 34 pharmacotherapeutic groups [7]. In 2020, an operationalization of the EU(7)-PIM list was carried out for the Portuguese reality, which consists of 184 PIMs; 178 correspond to active substances, five are drug classes, and one corresponds to the sliding scale therapeutic scheme used in insulins [16].

### 2.3. Statistical Analysis

The statistical analysis was performed with statistical software R (v4.1.2). All p values obtained ≤ 0.05 were considered statistically significant. The difference between the values obtained at hospital admission and discharge was calculated, and the dependent variables were categorized into 3 groups: no changes (reference category), increase, and decrease. A generalized linear model was developed for the dependent variables of multinomial type, performing bivariate analysis for each of the covariates and a multivariate analysis for each of the dependent variables. Odds ratios (OR) were obtained with 95% CI for the increase category and for the decrease category. The model was adjusted according to gender, patient age, total medication, days of hospitalization in the internal medicine service, and diagnoses.

A generalized linear model was built using negative binomial regression whose dependent variable is the PIMs at discharge, obtaining adjusted relative risks (RR) in 95% CI. The model was adjusted according to whether the patient had polypharmacy or not.

## 3. Results

### 3.1. Study Population Characteristics

A total of 662 older patients were admitted to the internal medicine ward in 2019, and 46 were excluded because their number of hospitalization days was less than 4. The median age of the 616 older patients was 85.0, and 51.6% were males. The median of hospitalization days was 12.00. A total of 3873 diagnoses were registered, 21.4% belonging to the group of diseases related to the circulatory system, 6.4% to endocrine, nutritional, and metabolic diseases, and 10.7% to respiratory system diseases. A detailed characterization of the included population can be found in a previous study [13].

### 3.2. Overall Medication Prescribed

In this study, we found that at admission, 6481 medicines were recorded, which increased to 7239 at discharge. Patients took a median of 10 medicines at admission and 11 at discharge. A prescription of five or more drugs was observed in 95.3% of older patients at admission and in 97.1% at discharge. (Table 1).

Multinomial regression used to obtain OR analysis revealed that the risk of having more prescribed drugs at discharge compared to admission is reduced by half in patients with infectious and parasitic diseases (OR 0.45; CI 0.24–0.81) and in patients with skin and subcutaneous tissue diseases (OR 0.52; CI 0.27–0.98). The risk increases in patients with diseases of the musculoskeletal system and connective tissue (OR 2.71; CI 1.08–6.76) and in patients with diseases of the genitourinary system (OR 1.51; CI 1.06–2.15).

### 3.3. Prevalence of PIM Prescription

According to the Beers criteria, 80.7% of older patients had at least one PIM at admission and 87.2% at discharge. When applying the STOPP criteria, 49.4% of older patients had at least one PIM at admission and 62.2% at discharge, and with the application of the EU (7)-PIM list adapted to the Portuguese reality, 51.3% of older patients had at least one PIM at admission and 70.3% at discharge. (Table 1).

From the analysis of the total number of PIMs prescribed, it was observed that by applying any of the criteria, the number of patients not taking any PIM on admission was higher than that at discharge. The opposite was found for patients taking two or more PIM, for which the number of patients was always higher at discharge than at admission, for all the applied criteria (Table 2).

### 3.4. Most Prescribed PIM at Admission and at Discharge

When analyzing the prescription of each PIM identified by each criterion, we observed that there are several PIMs that increased or decreased from admission to discharge (Table 3).

According to the Beers criteria, metoclopramide, a medicine that acts in the gastrointestinal tract, and quetiapine, sertraline, hydroxyzine, and haloperidol, medicines that act in the nervous system, were the PIMs with the highest increase from admission to discharge. On the other hand, the most-deprescribed PIMs were acetylsalicylic acid (a medicine that acts on blood), sulfamethoxazole and trimethoprim (an anti-infectious medicine), and tiapride (medicine that acts in the nervous system). Other medicines such as amiodarone, clemastine, desmopressin, midazolam, or mexazolam have a decrease of only one prescription from admission to discharge.

The application of the STOPP criteria revealed that the most increased PIMs were quetiapine, hydroxyzine, haloperidol, and morphine, medicines that act in the nervous system, and butylscopolamine, a medicine that acts in the gastrointestinal tract. On the other hand, the most-deprescribed PIM was captopril (medicine that acts in the cardiovascular system). Medicines such as tiapride (medicine that acts in the nervous system), and valsartan, ramipril, and enalapril (medicines that act in the cardiovascular system) had a decrease of two prescriptions. Other medicines have a decrease of only one prescription from admission to discharge.

Through the application of the EU (7)-PIM list, it was found that the most increased PIMs were bisacodyl, metoclopramide, and sitagliptin, medicines that belong to the alimentary tract and metabolism ATC group, followed by hydroxyzine and haloperidol, medicines that act in the nervous system. On the other hand, there was a deprescription of two propanol PIMs, and other medicines such amiodarone, clemastine, and phenytoin have a decrease of only one prescription from admission to discharge.

### 3.5. Factors Related to Changes in PIMs

Multinomial regression analysis revealed that the risk of PIMs increasing or decreasing at discharge according to the applied criterion were associated with gender, the total medicines that the patient took, and specific diagnoses (Table 4).

Negative binomial regression analysis showed associations between PIMs obtained with the application of each criterion and age, polypharmacy, and several health conditions (Table 5).

## 4. Discussion

This study found that the prescription of PIMs increased between hospital admission and discharge for older patients, in a sample of patients hospitalized in an internal medicine ward.

Depending on the criteria used, other factors were found, for example, females showed an increased risk of PIMs at discharge for PIMs classified by the Beers criteria and the EU(7) PIM List. According to Tian et al. (2021), females have an increased risk of using PIMs [17].

A study conducted in a hospital setting in Lithuania where the Beers 2015 criteria, the STOPP v2 criteria, and the EU(7)-PIM list were applied at hospital admission and discharge found that the percentages of patients with at least one PIM were 44.7–59.2%, 67.1–71.1%, and 69.7–72.4%, respectively [18], different values from those obtained in our study, although there was also an increase in PIMs between admission and discharge. A study in people with dementia and cognitive impairment where the Beers criteria were applied showed a significant decrease in PIMs between hospital admission and discharge (96% on admission and 87% on discharge) [19]. Another study where the STOPP criteria were applied showed that 39.3% of patients had been prescribed at least one PIM on admission, which increased to 46% on discharge, and a falls history increased the risk of having PIMs [20].

The increase in PIMs at discharge observed in our study suggests that in the hospital where the study took place, medication review may not yet be properly entrenched, or perhaps the prescription program implemented in the institution does not effectively help in PIMs identification. We must also consider that PIMs lists are tools developed to help the physician to detect PIMs, but the decision to prescribe a PIM or not belongs to the physician; perhaps the PIMs prescribed were the most adequate clinical decision for to the clinical situation of the patients.

Polypharmacy was found in 95.3% of patients at admission, which increased to 97.1% at discharge. According to Midão et al. (2018), polypharmacy is associated with older people with complex therapeutic regimens, and the existence of several comorbidities makes it necessary to prescribe several medicines, which may explain this increase in polypharmacy between admission and discharge [21].

Kable et al. (2019) also found high frequencies of polypharmacy (94% on admission and 90% on discharge) but, in contrast to our findings, they found a decrease in the frequency of polypharmacy between admission and discharge, and suggest that the optimization of pharmacotherapeutics could be carried out and/or improved during hospitalization [19]. Argoullon et al. (2018), also observed that the number of polymedicated patients is higher at discharge than at admission [22]. We found that patients with diseases of the musculoskeletal system, connective tissue, or genitourinary system have a higher risk of using multiple medicines at discharge. Our data also suggest that polypharmacy is associated with an increased risk of PIMs at discharge. Polypharmacy has been associated with ADR and hospital admissions [21,22,23].

We also found that haloperidol was one of the five most-prescribed PIMs at admission and discharge common to all three criteria, which shows that during hospitalization in the internal medicine service, haloperidol is often prescribed to older patients. Haloperidol is an antipsychotic drug that can help relieve some disorders, delusions, or hallucinations in schizophrenia situations. Haloperidol is also used in older patients with signs of agitation or aggression, which may explain the high consumption of this drug in the study population. Studies indicate that delirium is associated with substantial morbidity and mortality rates in hospitalized older patients, which is becoming a growing problem due to increased life expectancy, and haloperidol is usually used for the treatment of delirium [24,25,26]. However, haloperidol is for neuroleptic malignant syndrome, dystonia, extrapyramidal disorders, neutropenia, etc. in older patients, and it should be used carefully, monitored closely, and deprescribed as appropriate to minimize the risk of ADR [27].

Older patients are at particular risk of PIMs; PIM frequencies from 24% to 92% were reported [11,28,29,30,31,32], and these frequencies are different according to the applied criterion of the PIMs classification tool.

In this study, with the application of the Beers criteria, it was found that 80.7% of the patients had been prescribed at least one PIM on admission and 87.2% at discharge. After applying the STOPP criteria, it was found that 49.4% of patients had been prescribed at least one PIM at admission and 62.2% at discharge. The EU(7)-PIM list revealed that 51.3% of patients had been prescribed at least one PIM on admission and 70.3% at discharge. The diverging number of PIMs observed after the application of each PIMs tool can be related to the different medicines/drug classes and also to the different requirements for each medicine to be considered a PIM [7,14,15]. These differences in the perception of the PIMs detection tools can be associated with a lack of concordance among the different applied criteria, which, in turns, makes the implementation of measures to prevent PIMs difficult [11].

As previously reported by Perpetuo et al. [11], the poor concordance among criteria observed in this study suggests the need to re-design the existing criteria for medical ward inpatients.

The results of this study showed that PIMs identified with the application of the Beers 2019 criteria are higher at admission or discharge than those identified with the application of the STOPP v2 criteria and the EU(7)-PIM list. Despite this, the PIMs identified with the application of the EU(7)-PIM list increased by 19% from admission to discharge, while the PIMs obtained with the application of the STOPP criteria increased by 12.8% and the PIMs obtained with the application of the Beers 2019 criteria only increased by 6.5%.

Considering the need for deprescribing to reduce polypharmacy [33], it becomes important to reduce the number of prescribed medicines, especially if PIM prescriptions occur [19,34]. However, as previously described, the multiple comorbidities of internal medicine inpatients associated with the lack of evidence for the use of some medicines in older adults and the need to treat/stabilize the cause of the hospitalization favors the prescription of multiple drugs, including PIMs [13].

This study had some limitations, mainly related to lack of representativeness, as in this study, we included only one medical service of one hospital, and because of that, the results found cannot be generalized. However, all older patients admitted to this internal medicine ward who met the inclusion criteria were included in the study.

## 5. Conclusions

In this study, we found a high prevalence of PIMs at admission and discharge, and the prevalence of PIMs at discharge was higher than those obtained at admission, suggesting the need for studies to assess factors and barriers that may be associated with this high prevalence, as well as the development of interventions and measures that optimize the prescription of medicines to the older population.

The poor concordance between PIMs criteria observed also reinforces the need to optimize and validate criteria adapted to the hospital reality and specifically to different specialties, and make them easy to use during clinical practice.

## Figures and Tables

**Table 1:** Medications at admission and discharge.

Medication Outcome	Measure	Admission n = 6481	Discharge n = 7239
Number of medicines	median (Q1–Q3) range (min-max)	10 (8–13) 0–27	11 (9–14) 1–27
Polypharmacy (≥5 meds)	no yes	29 (4.7%) 587 (95.3%)	18 (2.9%) 598 (97.1%)
PIMs Beers criteria (at least one)	no yes	119 (19.3%) 497 (80.7%)	79 (12.8%) 537 (87.2%)
PIMs STOPP criteria (at least one)	no yes	312 (50.6%) 304 (49.4%)	233 (37.8%) 383 (62.2%)
PIMs EU (7)-PIM list (at least one)	no yes	300 (48.7%) 316 (51.3%)	183 (29.7%) 433 (70.3%)
n—number of medicines			

**Table 2:** Number of PIMs, according to the Beers and the STOPP criteria and the EU (7)-PIM list.

Frequency of PIMs	Tool					
	Beers 2019 Criteria		STOPP v2 Criteria		EU (7)-PIM List	
	Admission	Discharge	Admission	Discharge	Admission	Discharge
0	119 (19.3%)	79 (12.8%)	312 (50.6%)	233 (37.8%)	300 (48.6%)	183 (29.7%)
1	238 (38.6%)	190 (30.8%)	174 (28.2%)	190 (30.8%)	204 (33.1%)	205 (33.2%)
2	147 (23.8%)	162 (26.3%)	88 (14.3%)	119 (19.3%)	80 (13.0%)	152 (24.6%)
3	68 (11.0%)	116 (18.8%)	30 (4.9%)	44 (7.1%)	24 (3.9%)	50 (8.1%)
4	28 (4.5%)	47 (7.6%)	7 (1.1%)	24 (3.9%)	5 (0.8%)	19 (3.1%)
≥5	16 (2.6%)	22 (3.6%)	5 (0.8%)	6 (1.0%)	3 (0.5%)	7 (1.2%)

**Table 3:** PIMs’ increase and decrease from admission to discharge according to the Beers and the STOPP criteria and the EU (7)-PIM list.

Variation Type	Beers 2019				STOPP v2				EU (7)-PIM List			
	Drug	PIMs Admission (n)	PIMs Discharge (n)	PIMs Variation	Drug	PIMs Admission (n)	PIMs Discharge (n)	PIMs Variation	Drug	PIMs Admission (n)	PIMs Discharge (n)	PIMs Variation
Increase	Metoclopramide	105	148	43	Quetiapine	36	66	30	Bisacodyl	30	95	65
	Quetiapine	36	66	30	Hydroxyzine	13	40	27	Metoclopramide	105	148	43
	Sertraline	6	33	27	Haloperidol	84	109	25	Sitagliptin	7	42	35
	Hydroxyzine	13	40	27	Morphine	3	24	21	Hydroxyzine	13	40	27
	Haloperidol	84	109	25	Butylscopolamine	3	15	12	Haloperidol	84	109	25
Decrease	Acetylsalicylic acid	58	50	8	Captopril	46	37	9	Propranolol	6	4	2
	Sulfamethoxazole and Trimethoprim	5	0	5	Tiapride	10	8	2	Amiodarone	17	16	1
	Tiapride	10	8	2	Valsartan	4	2	2	Clemastine	2	1	1
	Clemastine	3	2	1	Enalapril	2	0	2	Phenytoin	1	0	1
	Amiodarone	17	16	1	Ramipril	25	23	2	-	-	-	-

**Table 4:** Factors associated with categories of increased or decreased PIMs.

PIM Tool	Variable	Adjusted OR (95%CI) Category	
		PIMs Increase	PIMs Decrease
Beers 2019 criteria	Females	0.93 (0.55−1.57)	1.58 (1.07−2.33) *
	Total medicines per patient	1.14 (1.09−1.19) *	1.09 (1.05−1.13) *
	I00-I99, diseases of the circulatory system	1.30 (1.08−1.57) *	1.15 (0.99−1.33)
	K00-K95, diseases of the digestive system	2.05 (1.31−3.2) *	1.49 (1.03−2.17) *
STOPP v2 criteria	Total medicines per patient	1.12 (1.06−1.17) *	1.1 (1.06−1.13) *
	K00-K95, diseases of the digestive system	1.54 (1.01−2.36) *	1.02 (0.71–1.47)
	R00-R99, abnormal symptoms, signs, and clinical and laboratory findings not classified elsewhere	1.14 (0.76−1.7)	1.33 (1.01−1.73) *
EU(7)-PIM list	Females	1.21 (0.66−2.2)	1.48 (1.01−2.18) *
	Total medicines per patient	1.14 (1.08–1.2) *	1.11 (1.07−1.15) *
	N00-N99, diseases of the genitourinary tract	0.97 (0.67−1.4)	0.75 (0.59−0.97) *

* *p* < 0.05.

**Table 5:** Factors associated with PIM prevalence at discharge.

PIMs Tool	Variable	Adjusted RR(95%CI)	*p*-Value
Beers 2019 criteria	Polypharmacy	1.83 (1.18–3.02)	0.0111
	F00-F99, mental, behavioral, and neurodevelopment disorders	1.18 (1.04–1.33)	0.0077
	I00-I99, diseases of the circulatory system	1.09(1.05–1.14)	<0.0001
STOPP v2 criteria	75 to 84 years	0.77 (0.6–0.98)	0.0363
	I00-I99, diseases of the circulatory system	1.07 (1.01–1.14)	0.0195
	V00-Y99, external causes of morbidity	1.47 (1.05–2.03)	0.0231
EU (7)-PIM list	85 years or older	0.77 (0.63–0.94)	0.0095
	Polypharmacy	3.34 (1.69–7.89)	0.0018
	D50-D89, blood and blood-forming organs diseases involving immunological mechanism	1.19 (1.04–1.36)	0.0096
	I00-I99, diseases of the circulatory system	1.07 (1.01–1.12)	0.0149
	N00-N99, diseases of the genitourinary tract	0.88 (0.80–0.97)	0.0089

## Data Availability

Data can be accessed upon request to the authors.
